# Intra-Abdominal and Retroperitoneal Benign Lipomatous Tumors—An Extremely Rare Mimic of Liposarcoma and its Diagnostic Challenge

**DOI:** 10.1177/10668969231167511

**Published:** 2023-05-02

**Authors:** Faizan Malik, Andrew W. Allbee, Paul J. Zhang

**Affiliations:** 1 Department of Pathology and Laboratory Medicine, Perelman School of Medicine, 21798 Hospital of the University of Pennsylvania, Philadelphia, PA, USA

**Keywords:** lipoma, liposarcoma, retroperitoneum, MDM2, CDK4

## Abstract

*Background.* Lipomas are common superficial soft tissue tumors of mature adipocytes. In contrast, well-differentiated/dedifferentiated liposarcoma typically presents in the retroperitoneum as large masses. We provide clinicopathologic and follow-up details of 9 retroperitoneal/intra-abdominal benign lipomatous tumors (BLT) and discuss the utility of ancillary fluorescence in situ hybridization (FISH) in distinguishing from their malignant counterparts. *Design.* Clinicopathologic details and histology of 9 intra-abdominal and retroperitoneal lipomas were studied along with ancillary CD10 immunohistochemistry (IHC) and FISH for *MDM2* and *CDK4* amplification. *Results.* There were 6 females and 3 males. Median age at diagnosis was 52 years (range 36-81 years). Seven were identified incidentally and 2 presented with primary complaints. On imaging, 7 were considered suspicious for liposarcoma. Grossly, the tumors ranged from 3.4 to 41.2 cm (median 16.5 cm). Histologically, all cases showed well-differentiated BLT, further classified as lipoma (n = 7; 1 with metaplastic ossification, 2 with prominent vessels, and 4 ordinary lipomas) and lipoma-like hibernoma (n = 2)—the latter 2 showed intramuscular lesions with interspersed brown fat. CD10 IHC showed strong staining in the 2 hibernomas, whereas the staining was weak in the remaining. *MDM2* and *CDK4* amplification were negative by FISH in all. Follow-up (median 18 months) did not show recurrence on clinical or imaging evaluation. *Conclusion.* Retroperitoneal/intra-abdominal BLT are extremely rare and are indistinguishable clinically and radiographically from liposarcoma. This necessitates molecular confirmation even when the histology is convincingly benign, for a confident diagnosis. Our cohort shows that conservative excision without removal of abutted organs is sufficient in most cases.

## Introduction

Lipomas are common benign tumors of fully mature adipocytes that tend to present in the upper back, proximal extremities, and abdominal region as a superficial soft tissue mass. Rarely, these may present in the deeper subcutaneous tissue or in viscera.^1^ In 2009, Macarenco et al presented the largest series of 19 well-differentiated adipocytic tumors^2^ prior to which, and because of the overwhelming predilection of well-/de-differentiated liposarcoma (WD-/DD-LPS) at this site, large fatty tumor of the retroperitoneum would be presumed to be liposarcoma. Using cytogenetic and molecular genetic characteristics, they confirmed that not all lipomatous tumors in the retroperitoneum are liposarcomas and a small subset represents benign lipomas. These tumors are, however, still uncommon and pose diagnostic challenges clinically and histologically. In this retrospective study, we describe the clinicopathologic features and provide follow-up information on 9 abdominal and retroperitoneal lipomas that we diagnosed since 2009 with the help of fluorescence in situ hybridization (FISH) assay for *MDM2* amplification. We further discuss differential diagnoses and utilization of FISH techniques for a confident diagnosis.

## Materials and Methods

The study was approved by the institutional review board. Specimens were retrieved from the electronic database of the hospital laboratory using the terms “retroperitoneal,” “intra-abdominal,” and “lipoma” with the exclusion of “liposarcoma,” diagnosed after 2009 up to 2022. Concomitantly, a search was performed to obtain the number of lipomas diagnosed in our lab during the same timeframe. An extended search for tumors prior to 2009 using a variety of terminology did not show additional cases in the archives.

Clinicopathologic and follow-up information were obtained from the medical records and from the referring physicians. One section per centimeter of the tissue was submitted, according to the grossing protocol in our laboratory. The diagnosis was confirmed based on morphologic, immunohistochemical, and molecular (FISH) features. Hematoxylin and eosin-stained sections of formalin-fixed paraffin-embedded (FFPE) blocks were reviewed.

As CD10 has recently shown to be selectively highly expressed in hibernomas,^3^ we retrospectively performed CD10 on all tumors to illustrate any difference in expression between tumors with and without brown fat. For these immunohistochemical studies, 4-μm-thick sections were cut from FFPE tissue blocks and mounted on positively charged slides. Sections were processed on a Bond-III automated staining system (Leica Biosystems), deparaffinized, and then subjected to heat-induced epitope retrieval. Sections were then stained with CD10 (RTU mouse monoclonal antibody; 56C6; catalog # PA0131; Leica Biosystems) on all. In 2 lesions, to exclude a spindle cell lipoma presenting at an unusual site or an atypical spindle cell lipomatous tumor,^4^ immunostaining for Retinoblastoma-1 antigen (RB1, clone 1F-8; 1:50; Thermo Fisher Scientific) and CD34 (RTU mouse monoclonal antibody; QBEND10; Leica Biosystems) were performed at review.

Prospectively, at the time of diagnosis, FISH for mouse double minute 2 (*MDM2*) was performed on all, and for cyclin-dependent kinase 4 (*CDK4*) on 5 tumors. Retrospectively, *CDK4*) FISH was performed on remaining 4 tumors at the time of this review. FISH for *MDM2* and *CDK4* gene amplification was performed using 4-μm FFPE tissue sections of the tumors using commercially available dual DNA probes. For *MDM2*, dual DNA probes targeting *MDM2* (12q15) and a centromeric region of chromosome 12 (SE12) (Kreatech Repeat-Free™ Poseidon™, Leica Biosystems) was used. The *MDM2* (12q15) gene region probe is directly labeled with PlatinumBright™550 (red). The centromeric12 control probe is directly labeled with PlatinumBright495 (green). To detect *CDK4* amplification, commercially available dual DNA probes for *CDK4* (12q13) and a Satellite Enumeration 12 (SE12) to centromeric region of chromosome 12 (Kreatech Repeat-Free™) were used. The *CDK4* (12q13) gene region probe is direct labeled with PlatinumBright™550 (red). The SE12 control probe is directly labeled with PlatinumBright™495 (green). In addition to the ratio of *MDM2* or *CDK4* copy signal against CEP12 or SE12 signal of more than 2.0, the morphologic feature of the amplified signals in cluster more than 6/cell was considered specific for WD-/DD-LPS. Immunohistochemical stain for MDM2 was not performed since its specificity and sensitivity is inferior to FISH. On the 2 cases with a question of spindle cell lipoma or atypical spindle cell lipomatous tumor, FISH for *RB1* (RB1/13q14; Vysis LSI 13, Abbott Laboratories) was performed.

## Results

### Clinical and Imaging Findings

Clinical, imaging, and follow-up information are summarized in Table 1. From a total of 1039 lipomas diagnosed at our institution, 9 (0.77%) were located in the abdominal cavity or retroperitoneum. Among these patients, 6 were females and 3 were males. The median age at identification of the masses was 52 years (range 36-81 years). Only 2 patients presented with a related complaint while the tumors were detected incidentally in 7 patients. Among the 2 with clinical symptoms, one patient noticed asymmetry of the abdomen and the other complained of progressive abdominal bloating and back pain. In the remaining 7 patients, the tumor was detected in 5 by imaging for unrelated reasons; in one patient, the tumor was noticed perioperatively during surgery for a urinary bladder cystocele repair, while in the other, the primary physician noticed a lump on abdomen palpation in an annual visit and was subsequently imaged. The comorbidities that prompted imaging in those 5 patients included: primary biliary cirrhosis, lumbar radiculopathy, surveillance after surgery for sigmoid colon adenocarcinoma with lymph node metastasis, flank pain due to renal stones, and follow-up for recently diagnosed endometrial carcinoma. In the patient who had colorectal surgery 4 years’ prior, no soft tissue masses were identified perioperatively or in the interim.

**Table 1.** Clinicopathologic Characteristics of Patients.

Patient	Age (years)/Sex	Clinical history	Imaging findings and impression	Operative procedure	Gross size (largest dimension)	*MDM2* FISH	*CDK4* FISH	Follow-up (months)
1	81/Female	Recurrent UTI; cystocele and diverticulum of urinary bladder	Not visualized on imaging, incidentally found perioperatively	Complete excision	3.4 cm	Neg	Neg	DOC
2	64/Female	Cirrhosis; PBC; Liver and kidney transplant; mass detected during imaging for other diseases	15.8 cm circumscribed, fat attenuation mass with internal septation in the right retroperitoneum with mild mass effect on the inferior pole of right kidney; liposarcoma	Complete excision after separation from Gerota's fascia	26 cm	Neg	Neg	ANER (129); followed by imaging for 92 months (for transplant), then clinically
3	36/Male	Self-visualized asymmetry of right lower abdominal wall	10 cm encapsulated and lobulated fat-containing mass filling the right lower quadrant extending to below the right kidney and reaching up to the anterior abdominal wall and iliopsoas musculature; lipoma	Complete excision of mass after separation from iliopsoas musculature	13.1 cm	Neg	Neg	ANER (20); followed by imaging
4	59/Male	Lumbar radiculopathy with incidental detection of “fatty” mass in abdomen	16 cm, minimally complex fat-containing lesion in the anteromedial aspect of left psoas muscle with lobulated, partially calcified focus along with the upper aspect; “atypical lipoma” versus WDLS	Complete excision after separation from musculature	16.5 cm	Neg	Neg	ANER (21); followed by imaging for 6 months, then clinically
5	44/Female	Abdominal bloating and back pain	20 cm mass in the left retroperitoneum, predominantly fatty with multiple septations and encasing the left kidney with mass effect; suspicious for well-differentiated liposarcoma	Conglomerate of tumors completely removed in piecemeal	41.2 cm; 2 additional resected portions—14 cm and 14.2 cm	Neg	Neg	ANER (20); followed clinically
6	52/Male	Sigmoid colon adenocarcinoma metastatic to lymph nodes, mass detected on surveillance imaging 4 years post-colectomy	12 cm, slowly growing retroperitoneal mass with variably thick encapsulation of up to 3 mm and suspicious solid component, displacing the left kidney anteriorly; lipoma with low suspicion for liposarcoma	Several separate tumors in the abdomen visualized perioperatively, incomplete removal for debulking	13.2 cm; separate pieces 10.8 cm in aggregate	Neg	Neg	ANER (16); followed by imaging
7	40/Female	Flank pain due to renal stones; mass discovered incidentally on imaging for renal stones, previous 2 c-sections, cholecystectomy (interval unknown)	16.7 cm mass in right lower pelvis with displacement of uterus and bladder to the left and superior displacement of the right ovary, suspicious for WDLS versus “atypical” lipoma	Complete excision	19.2 cm; smaller excised peritoneal nodule showed a benign multicystic mesothelioma	Neg	Neg	Recent surgery (6-month follow-up); ANER by imaging
8	70/Female	Recently diagnosed serous endometrial carcinoma, imaging revealed a large retroperitoneal mass.	14.6 cm fat-containing mass in the right pararenal space with enhancing solid component, suspicious for retroperitoneal liposarcoma	Partial resection	8.9 cm	Neg	Neg	Recent surgery (3 months follow-up)
9	68/Female	Right lower quadrant mass detected incidentally by PCP on physical examination; occasional nausea	15.8 cm mass within the lower abdomen and pelvis with several soft tissue septations; impinging on adjacent bowel; favored to be a low-grade liposarcoma	Partial resection	In multiple pieces, 15 cm in aggregate	Neg	Neg	ANER; presented after 15 months with ventral abdominal incisional hernia due to protrusion of residual lipoma; FISH for *MDM2* amplification was negative in the reexcision. Imaging prior to reexcision did not show growth of residual tumor

Abbreviations: FISH, fluorescence in situ hybridization; UTI, urinary tract infection; DOC, died of other cause(s); PBC, primary biliary cirrhosis; ANER, alive and no evidence of recurrence; WDLS, well-differentiated liposarcoma; PCP, primary care physician.

**Figure 1.**
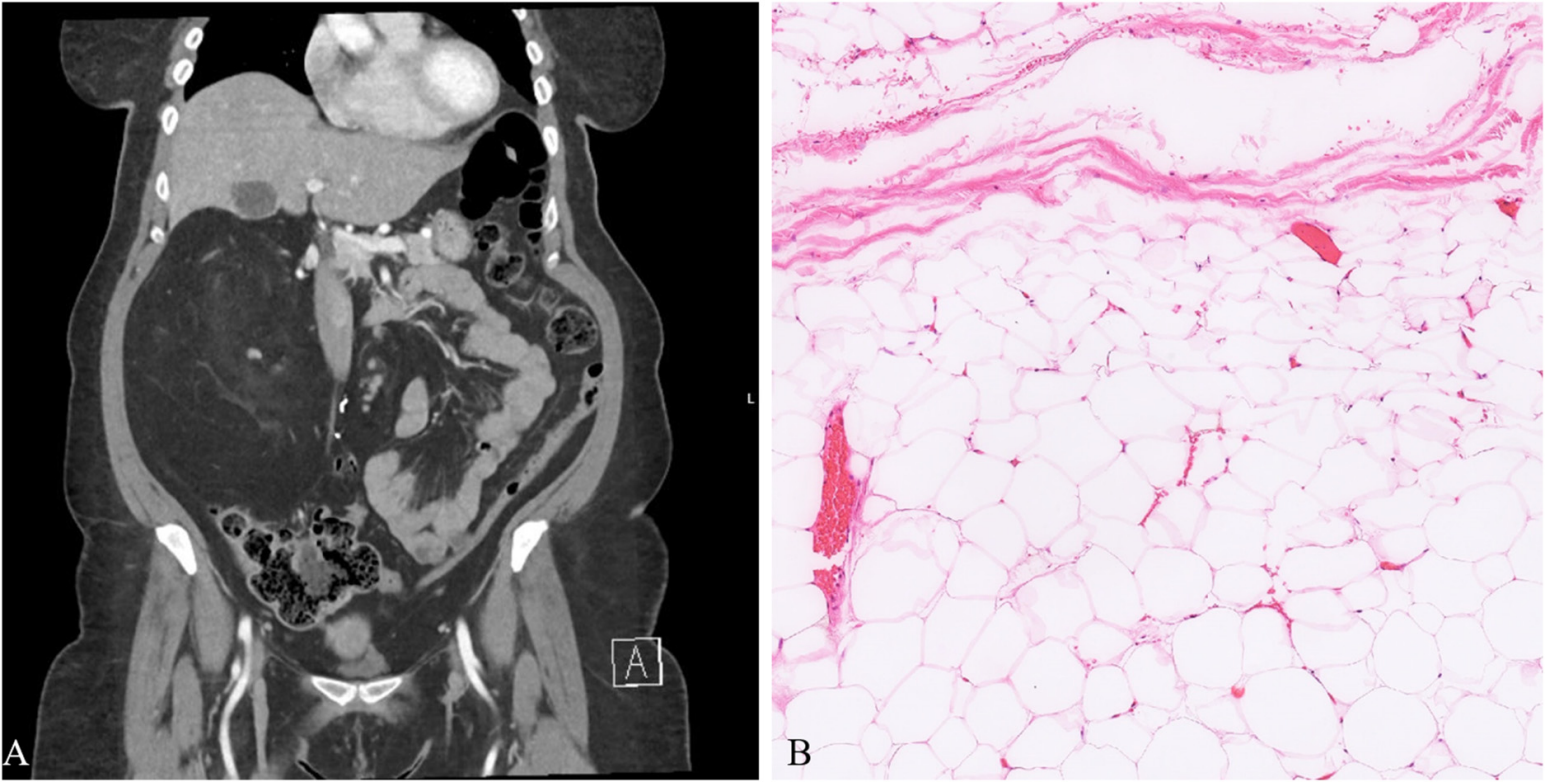
A, Computed tomography scan shows a large, 14.6 cm fat-containing mass in the right retroperitoneal, pararenal space with slight enhancement of a small solid component. (B) “Giant” lipoma arising in the retroperitoneum showing typical features of mature adipocytes with thin fibrous strands traversing through fat lobules (patient 5).

On computed tomography or magnetic resonance imaging, the masses were described as encapsulated of variable thickness, lobulated, complex, and with internal septations. The masses displaced the ipsilateral kidney or bowel in 7 examples. A suspicion of liposarcoma was raised in 7. In one tumor (patient 4), a lobulated, calcified focus was identified, which correlated histologically (see below). Two lesions showed encasement of iliopsoas muscle. Radiographic appearance of one example (patient 8) is shown in Figure 1A.

Follow-up was available for all patients. The median follow-up period was 18 months (range 3-129 months); follow-up for more than 12 months was available in 6 patients. There was no evidence of recurrence in any patient. Patient 9 presented to the surgeon 15 months after initial resection for ventral incisional hernia due to residual pelvic intra-abdominal lipoma; no clinical or radiographic recurrence was suspected.

### Gross Features

The median size of the tumors was 16.5 cm (range 3.4-41.2 cm). In 2 samples, there were additional fragmented pieces of the tumor with similar gross appearance. A thin, translucent capsule was present in all tumors. The cut surfaces of all tumors showed yellow, homogenous, lobulated appearances. There were no visible areas of hemorrhage or necrosis. Fibrous tissue was noted grossly in 3 tumors (patients 3, 5, and 6); however, this was not replacing the lobulated cut surface of the specimens. In patient 5, firm areas of calcification were also present. If needed, additional sections were submitted from any concerning areas. The small 3.4 cm lipoma (patient 1) perioperatively and grossly appeared distinct from lobulated peritoneal fat.

### Microscopic, Immunohistochemical, and FISH Features

**Figure 2.**
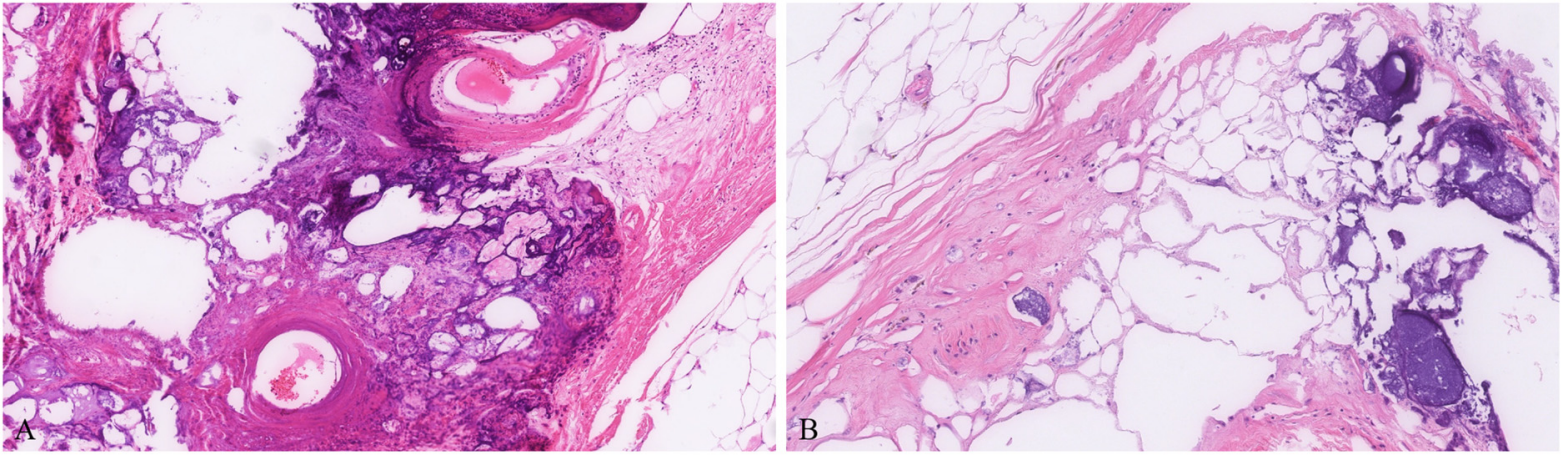
Rare tumors can show metaplastic ossification (A and B) and can be worrisome for dedifferentiation. In this example (patient 4), calcification and ossification were present near areas of fat necrosis (B).

**Figure 3.**
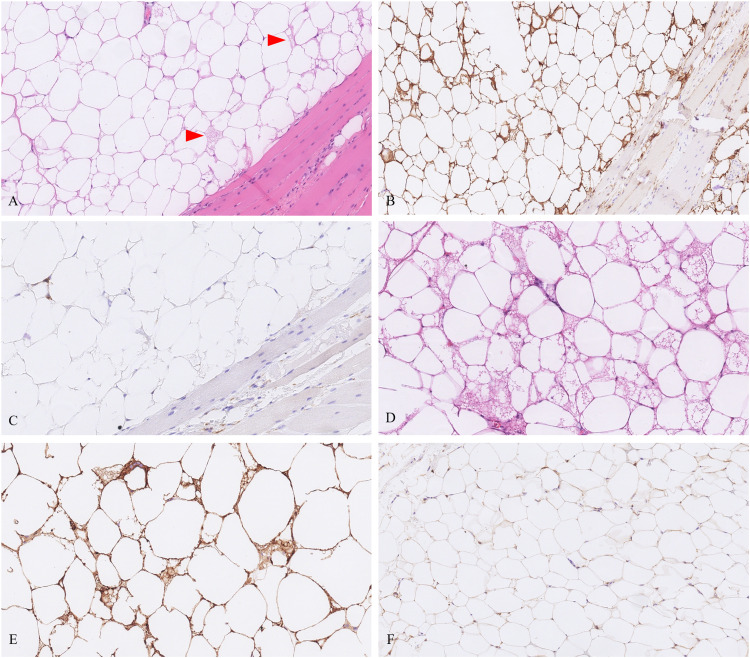
A, Tumor from patient 3 showed an intramuscular retroperitoneal lipoma-like hibernomas with scattered rare brown fat cells (arrowheads). (B) CD10 showed strong staining in this example. (C) CD68 is shown in the same areas, which helps to discriminate from histiocytes. (D) The tumor from patient 6 showed prominent brown fat cell population with strong CD10 staining (E) as compared to negative or faint positivity in ordinary retroperitoneal lipoma without brown fat (F).

**Figure 4.**
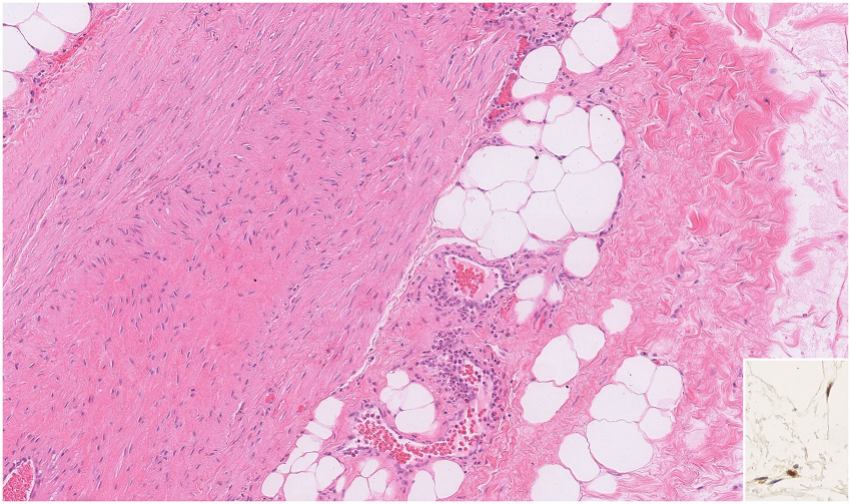
Lipoma with prominent vasculature. A thick vessel wall with intervening adipocytes may prompt additional workup to exclude other differential diagnoses. In such examples, spindle cell lipoma or atypical spindle cell lipomatous tumor can be considered in the differential which was excluded based on morphology and retained RB1 staining (inset). In addition, CD34 was negative (not shown).

Microscopic sections of all 9 tumors showed nearly identical morphology with only slight variability. All tumors were thinly encapsulated. All neoplasms were composed of mature adipocytes with distinct cell boundaries and peripherally placed thin nuclei (Figure 1B). One tumor showed prominent osseous metaplasia with focal myxoid change (Figure 2). In 2 tumors, a prominent admixture of brown fat cells was noted (patients 3 and 6; Figure 3). Both these lesions also showed entrapped skeletal muscle, compatible with intramuscular lipoma-like hibernoma and were reclassified as such. Four tumors showed multiple thick-walled blood vessels and traversing fibrous bands. In 2 of those 4 tumors (patients 2 and 5), the extent of vessel wall thickness (Figure 4) prompted the reviewing pathologists to perform ancillary testing at the time of diagnosis to rule out differential diagnoses such as angiomyolipoma or lipoleiomyoma but were not supported by additional testing and hence were ultimately classified as lipomas with prominent vessels at the time of diagnosis. No atypical hyperchromatic stromal cells were identified in any specimen.

**Figure 5.**
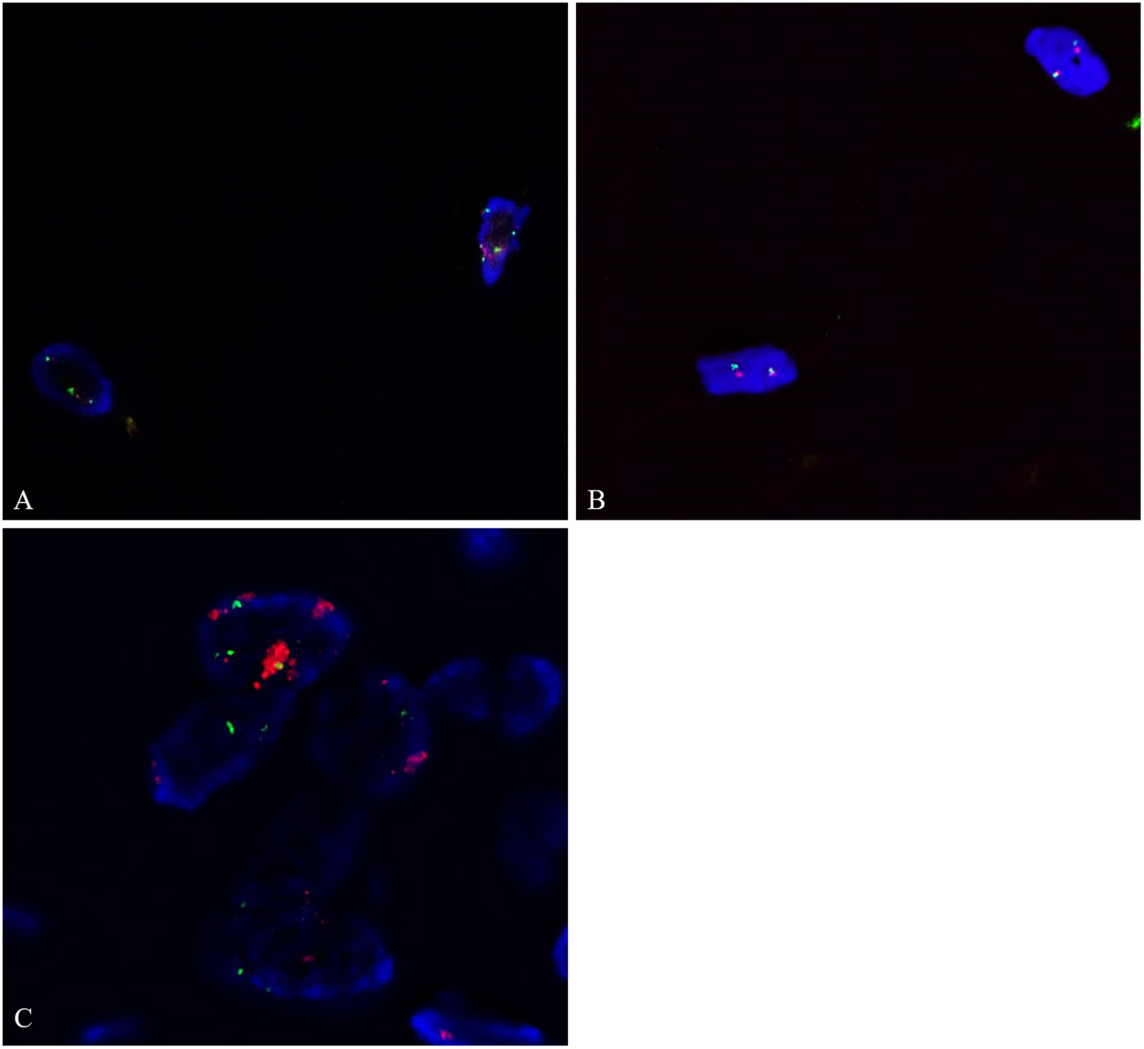
Fluorescence in situ hybridization for *MDM2* (A) and *CDK4* (B) shows a lack of amplification. (C) An example of dedifferentiated liposarcoma with *MDM2* amplification is shown for comparison (red signal in A and C, *MDM2* probe; red signal in B, *CDK4* probe; green signal in A-C, CEP12 probe).

CD10 immunohistochemistry (IHC) was performed to detect any variation of staining between the lipomatous tumors with and without the hibernomatous components (Figure 3B, E, and F). CD10 showed diffuse and robust positivity in tumors from patients 3 and 6. It was negative to very faintly positive in 3 tumors and showed mild to moderate reactivity in 4 tumors. The staining was located predominantly in the cell membranes with very rare cells positive in the stroma of the fibrous bands. For the 2 tumors with thick-walled blood vessels where spindle cell lipoma was considered in the histologic differential at the time of this review (patients 2 and 5), CD34 was negative and RB1 was retained in the adipocyte and stromal nuclei (Figure 4 inset). RB1 FISH on 1 of 2 tumors showed retained probe signals in more than 80% of the cells. In the other tumor, there was poor probe hybridization in the adipocytes as well as native endothelium, likely due to older archival tissue and hence was inconclusive. Even in the absence of the confirmatory FISH assay, the lack of supportive morphologic and immunohistochemical features was sufficient to exclude a spindle cell lipoma in both instances. FISH studies in all 9 tumors did not show amplification of *MDM2* or *CDK4* (Figure 5).

## Discussion

Lipoma is a common, benign soft tissue tumor of mature adipocytes that present in the superficial subcutis of the extremities and body. It typically presents as a painless mass with up to 5% of patients having multiple lesions and a small number having an association with PTEN hamartoma syndrome. Although it is considered mainly a tumor of soft tissue and skeletal muscle, lipomas are also reported in unusual sites such as the airways, gastrointestinal tract, and head and neck region, including intracranial sites and salivary glands.^1,5-8^ Lipomas are usually less than 5 cm in size but rarely can grow to large sizes. In their review, Sanchez et al suggested the prefix of “giant” for any lipoma that exceeds the 10-cm cutoff.^9^ Their review, however, did not include any intrabdominal or retroperitoneal lipomas.

When arising in the subcutaneous soft tissue in small size, the diagnosis is straightforward. Ruling out other possibilities becomes necessary when lipomas present in unusual sites or if they are remarkably large. Lipomas have been recognized to occur in the retroperitoneum but prior to, and since, Macarenco et al's large series,^2^ it is only described in case reports of unusual presentations,^10-19^ such that additional experience in clinical–pathological findings and differential diagnostic considerations are difficult to find. In this study, we describe the clinicopathologic features of 9 additional abdominal cavity lipomas that were histologically and molecularly distinguished from WD-LPS.

Nearly all patients presented within the fourth to sixth decades. In contrast to previous larger descriptions,^2^ the masses in most patients in our cohort were detected incidentally on imaging to monitor co-morbidities. When symptomatic, patients presented with visible abdominal asymmetry and bloating similar to that of WD-/DD-LPS. Given the large potential space of the retroperitoneum, the asymptomatic presentation is conceivable. As expected, symptoms result from the massive size and impingement on adjacent structures, including urinary urgency and frequency, bloating, nausea, and constipation.^17^ Rarely, these tumors present in pregnancy.^13,16,20^ On imaging, the possibility of liposarcoma was raised in 7 out of 9 patients because of the alarmingly large size and complexity imparted by the fibrous septae. In one patient, the multilobulated mass encased the kidney, further convincing for a liposarcoma. Origin from the iliopsoas muscle was detected radiographically in 2 patients (histologically both were intramuscular lipomas).

Microscopically, typical features of lipoma were appreciated in each instance. These included thin lobules of mature adipocytes, traversed by thin fibrous bands, with an outer fibrous capsule. Two tumors in our cohort showed a smaller number of brown fat cells, signifying that these were in fact lipoma-like hibernomas. Hibernomas are slow-growing benign tumors and are usually present in the subcutis. We hypothesize that the empty retroperitoneal space likely allowed these hibernomas to grow and mature, ultimately demonstrating lipoma-like morphology. Recently, Gjorgova-Gjeorgjievski et al showed that CD10 is consistently expressed in hibernomas.^3^ This was replicated in both retroperitoneal lipoma-like hibernomas where CD10 showed robust and diffuse expression within adipocyte membranes. Our experience with CD10 was slightly different than the abovementioned series^3^ in the sense that our comparison group of intra-abdominal lipomas (rather than ordinary extremity lipomas) did show some CD10 positivity, albeit never expressing the same intensity as the 2 hibernomas.

The differential diagnoses of lipomas in the retroperitoneum include WD-/DD-LPS, renal, or extrarenal angiomyolipoma (PEComa), and lipoleiomyoma—WD-/DD-LPS holding the most diagnostic significance and challenge. WD-LPS is a malignant adipocytic lesion with a predilection for the retroperitoneum. The lipoma-like (adipocytic) variant of WD-LPS closely mimics its benign counterpart. Although the different subtypes (inflammatory, sclerosing) often coexist, the presence of just the adipocytic component in a biopsy can be challenging. Features that aid in distinction include substantial variation in cell size with the presence of nuclear atypia in fat or stromal spindle cells.^21^ Progression of WD-LPS results in dedifferentiation, characterized by an abrupt transition to nonlipogenic sarcoma, in most instances to a high-grade type.^22-24^ Both WD-LPS and DD-LPS are cytogenetically characterized by the formation of supernumerary ring chromosomes, forming via an unknown mechanism, that contains amplified sequences in chromosome regions 12q14-q15.^25^ This results in amplification of *MDM2* gene that drives oncogenesis in liposarcoma.^25,26^ Several other genes are co-amplified in the amplicon that includes *CDK4* (most common), *TSPAN31*, *HMGA2*, *YEATS4*, *CPM*, and *FRS2*.^27^ MDM2 protein is an inhibitor of p53 transcriptional activity; gene amplification results in loss of p53 tumor suppressor function, leading to oncogenesis.^28^ This genetic alteration can be detected by FISH as well as IHC targeting the nuclear-localized protein.^29,30^ Recently, multiplex ligation-dependent probe amplification technique was also described by Creytens et al to detect *MDM2*/*CDK4* amplifications on FFPE with the high concordance with FISH.^31^ Given the relatively insensitive nature of immunohistochemical studies in differentiated lipomatous tumors, FISH is considered the more reliable and cost-effective option compared to other molecular tests.^29,30^ Sirvent et al also note that IHC alone is often insufficient to solve diagnostic problems.^29^ Further, MDM2 immunostain can show dim staining in histiocytes in traumatized lipomas, an important but underrecognized pitfall.^32^ In our laboratory, we rely solely on FISH detection for *MDM2* amplification. Apart from confirming the morphologic impression, this test is warranted for any “problematic” fatty tumors meeting any criteria described before.^33^ In all our patients, the clinical and radiographic suspicion of WD-LPS was high due to the location and unusually large size. Microscopically, however, the lack of cell size variation and absence of atypical stromal cells supported the benign nature. *MDM2* and *CDK4* amplification was absent by FISH in all tumors, thus underscoring its utility for a confident diagnosis.

While discussing WD-/DD-LPS, 2 other important, albeit rare, situations warrant attention—first, hibernomas that morphologically mimic atypical lipomatous tumor/WD-LPS^34^ and second, WD-/DD-LPS with hibernoma-like histology.^35^ In both instances, careful microscopic search for atypical stromal cells or nuclei and areas of dedifferentiation is critical. In addition, ancillary tests for MDM2 overexpression or *MDM2* amplification aid in distinction.^34,35^ Uncoupling protein 1 (UCP-1) is a recently described protein transporter and is considered a sensitive and specific marker of brown fat differentiation when compared to white/abdominal fat.^36^ Further, Kojima et al tested UCP-1 on their cohort of liposarcomas with brown fat differentiation and found that UCP-1 is expressed in these foci,^35^ which could be very useful to confirm brown fat differentiation when needed.

In contrast to WD-/DD-LPS, the genetic basis of some lipomas is attributed to translocations of *HMGA2* (High Mobility Group AT-Hook 2) gene to various partners (*LPP*, *CXCR7*, *EBF*, *LHFP*, and *NFIB*).^37-39^ HMGA2 acts as a transcriptional regulating factor and rearrangements result in deregulated activity. Although a commercial *HMGA2* FISH break apart probe is widely available, its utility in diagnosing ordinary lipoma is quite limited. In fact, it is more desirable to detect the absence of *MDM2* amplification^40^ to exclude liposarcoma. *HMGA2* translocations were reported by Macarenco et al in 42% of their retroperitoneal lipomas.^2^ In our experience, the absence of *MDM2* amplification was sufficient to reach a confident diagnosis.

Few other tumors with lipomatous elements in the area warrant mention for differential diagnosis, particularly when only lipomatous elements present in small core biopsy material. In addition to histology, radiologic/imaging correlation is also very important in the diagnosis of retroperitoneal lipoma against these tumors with lipomatous elements in small biopsy material. Angiomyolipoma is a benign mesenchymal tumor belonging to a family of lesions characterized by the proliferation of perivascular epithelioid cells, termed PEComa. These tumors have a wide anatomic distribution and can present in renal or extrarenal sites.^41-43^ These tumors are composed of a varying quantity of adipose tissue, spindled and epithelioid smooth muscle cells, and thick-walled blood vessels. A unique feature of this tumor category is the coexpression of smooth muscle and melanocytic markers, including HMB-45, Melan-A, MITF, SMA, desmin, and caldesmon.^42,44^ “Fat-rich” angiomyolipomas have been described, defined by more than 75% of tumor composed of mature fat.^45^ In a subset of our cases, we found thick-walled blood vessels with associated fibrous bands in the lobules of adipocytes. In some foci, these spindle cells appeared to emanate from the vessel wall, reminiscent of angiomyolipoma. However, both markers of smooth muscle and melanocytic differentiation were absent which aided in ruling out angiomyolipomas. A subset of PEComas is driven by *TFE3* fusions with corresponding nuclear TFE3 expression. In females, a large retroperitoneal fatty mass would also generate the differential of a uterine lipoleiomyoma. These are uncommon mesenchymal tumors that are primarily present in the uterus, however, rare extrauterine examples also exist.^46,47^ In the largest reported series by Wang et al, the tumors were mostly small but did occasionally reach up to 35.5 cm.^46^ Eight out of 50 tumors in their series showed adipocytes comprising 76% to 100% of the lesion. “Fat-rich” lipoleiomyoma was in the differential with angiomyolipoma in the same 2 specimens in our series. As mentioned earlier, negative muscle markers were supportive of a pure adipocytic tumor. PNL2 is a novel antibody that has shown high sensitivity and specificity for angiomyolipoma and PEComa,^48^ although its utility in pure adipocytic tumors has yet to be established. In any case, the differential diagnosis is usually not difficult in excision specimens when other nonlipomatous elements are also present in these tumors.

Spindle cell lipoma arising in an unusual location or an atypical spindle cell lipomatous tumor^4,49,50^ can be considered if the morphological features are supportive, especially when these features are dominant in a needle core biopsy. In such situations, a combination of CD34 with RB1 IHC is useful for confirmation or exclusion; RB1 FISH is a complementary or alternative tool. These RB1-altered tumors are considered to lie on a spectrum^50^ and so far have shown benign behavior with a small risk of recurrence in the latter.^4^

Although common in subcutaneous lipomas, fat necrosis was only seen in one retroperitoneal lipoma because of the concealed nature and the extremely low probability of mechanical trauma. Nevertheless, histiocytes can simulate lipoblasts (pseudolipoblasts) and can create confusion. As alluded to earlier, MDM2 immunostain can show spurious positivity and should not be overcalled.^32^ One tumor in our cohort showed marked chondrous and osseous metaplasia. These changes, although expected in long-standing lipomas, are rarely reported in giant lipomas.^51^ In fact, the presence of osseous and chondroid metaplasia in a retroperitoneal fatty tumor is more concerning for DD-LPS, than a lipoma. In that scenario, however, the osteocartilaginous elements would be expected to show clear-cut malignant features.^52,53^

In summary, our series provides another cohort confirming the presence of lipomas in the retroperitoneum, which were diagnosed prospectively with the help of *MDM2* FISH. Even though these are extremely rare (<1%), retroperitoneal and intra-abdominal lipomas could present with an ominous radiographic presentation, often escaping attention for a lengthy period. In our experience, they tend to achieve “giant” sizes as the empty retroperitoneum allows for growth without detection. The histologic features are typical of lipoma, except that rarely they may show the presence of brown fat or metaplastic changes. Molecular confirmation by FISH for lack of *MDM2* amplification is highly recommended to render a reliable diagnosis of retroperitoneal lipoma. Our findings support that adequate sampling (1 section per centimeter), careful microscopic evaluation, and absence of *MDM2* gene amplification (by FISH) are sufficient for a confident diagnosis, without the need for additional ancillary or molecular testing. Pathologists and clinicians should be aware that not all huge fatty tumors in the retroperitoneum are well-differentiated liposarcomas and the use of FISH assay on routine paraffin section for MDM2 amplification could effectively and reliably confirm the diagnosis of lipoma in the retroperitoneum.
